# Exploring the Pharmacological Mechanism of the Effective Chinese Medicines Against Gynecological Cancer Based on Meta-Analysis Combined With Network Pharmacology Analysis

**DOI:** 10.3389/fonc.2022.817772

**Published:** 2022-07-06

**Authors:** Ning Ren, Lulin Yu, Lihui Qian, Gewei Ye, Zhenzheng Zhu, Jieru Yu, Leitao Sun, Leyin Zhang

**Affiliations:** ^1^ Hangzhou TCM Hospital of Zhejiang Chinese Medical University (Hangzhou Hospital of Traditional Chinese Medicine), Hangzhou, China; ^2^ The First School of Clinical Medicine, Zhejiang Chinese Medical University, Hangzhou, China; ^3^ School of Basic Medical Sciences, Zhejiang Chinese Medical University, Hangzhou, China; ^4^ Department of Medical Oncology, The First Affiliated Hospital of Zhejiang Chinese Medical University (Zhejiang Provincial Hospital of Traditional Chinese Medicine), Hangzhou, China; ^5^ Department of Oncology, Hangzhou TCM Hospital of Zhejiang Chinese Medical University (Hangzhou Hospital of Traditional Chinese Medicine), Hangzhou, China

**Keywords:** gynecological cancer, chemotherapy, traditional Chinese medicine, meta-analysis, network pharmacology

## Abstract

This meta-analysis plus network pharmacology aimed to investigate whether traditional Chinese medicine (TCM) combined with chemotherapy is associated with more beneficial efficacy data in the treatment of gynecological cancer (GC). A total of 11 randomized controlled trials (RCTs) consisting of 863 GC patients were included. Results showed a better ORR (RR: 1.42, 95% CI: 1.18–1.71; *I* ^2^ = 21.4%; *p* *=* 0.282), DCR (RR: 1.13, 95% CI: 1.03–1.25; *I* ^2^ = 0.0%; *p* *=* 0.492), PD (RR: 0.27, 95% CI: 0.11–0.65, *p* *=* 0.003; *I* ^2^ = 0.0%, *p* *=* 0.930), and QOL (SMD: 0.85, 95% CI: 0.38–1.33, *p* *=* 0.005) and higher proportions of CD3^+^ T (WMD: 5.65, 95% CI: 4.23–7.08, *p* *=* 0.000; *I* ^2^ = 68.3%, *p* *=* 0.004), CD4^+^ T (WMD: 6.97, 95% CI: 5.35–8.59, *p* *=* 0.000; *I* ^2^ = 83.4%, *p* *=* 0.000), and the CD4^+^/CD8^+^ T ratio (WMD: 0.32, 95% CI: 0.23–0.42, *p* *=* 0.000; *I* ^2^ = 78.0%, *p* *=* 0.000). The number of adverse events (AEs) was significantly lower in the TCM + chemotherapy group. The active components and targets of 19 high-frequency Chinese medicines obtained from the meta-analysis were screened and explored in network pharmacology analysis. Also, a regulatory network of active components and targets, a core network and key genes, a diagram of protein interaction, network topology analysis, and gene body GO function and KEGG pathway enrichment analysis were performed. A total of 120 active components were identified. NPM1 and HSPA8 are the most critical target proteins in the core network of protein interaction. HSP90AA1 is the most important target protein in the TCM group. KEGG enrichment analysis showed that it was highly significant in the lipid and atherosclerotic pathways. Therefore, moderate evidence revealed that TCM plus chemotherapy has obvious advantages over chemotherapy alone in terms of tumor responses, QOL, peripheral blood lymphocyte levels, and fewer AEs in the treatment of GC. The potential important targets and core genes were displayed.

**Systematic Review Registration:**

www.crd.york.ac.uk/PROSPERO/, identifier CRD42021252500.

## 1 Introduction

More than 8.6 million global women are diagnosed with cancer per year, with approximately 1.3 million cases of gynecological cancer (GC) (1, 2). Despite the progressive enhancement in diagnosis and therapy strategies that has been increasing in the past decades, the prognosis of GC remains not to everyone’s satisfaction (3, 4).

The main treatment methods for gynecological tumors include surgery, chemotherapy, radiotherapy, or a combination of them. Chemotherapy is an important part of the treatment of gynecological tumors, but it will inevitably induce adverse effects such as deterioration, myelosuppression, and reduction. Take ovarian cancer (OC), for example. OC is the most common malignant tumor in gynecology, and the current standard treatment plan of which is postcytoreductive surgery combined with “paclitaxel + carboplatin” therapy. Also, emerging treatments such as poly(ADP-ribose) polymerase (PARP) inhibitor (5) and neoadjuvant chemotherapy (NACT) (6) are also popularly chosen to be applied in OC therapy, but how to optimize the clinical treatment plan, reduce the adverse effects of various treatments, and improve the quality of life of patients remains to be further explored.

In China, traditional Chinese medicine (TCM) has gained more and more attention for malignant tumors because of its beneficial effects, such as low toxicity and enhanced efficiency (7, 8). Furthermore, data have shown that TCM can significantly enhance the sensitivity to chemotherapeutic drugs and tumor-suppressing effects and relieve cancer-related adverse events (AEs) (9, 10). However, its clinical characteristics were still not well investigated. It remains uncertain whether TCM plus chemotherapy is more effective than chemotherapy alone for GC, which has not been systematically assessed.

Therefore, we aimed to drive this meta-analysis to investigate the efficacy and safety of combination therapy by using TCM plus chemotherapy compared with chemotherapy alone, in which way we can spot suitable prescriptions to improve patients’ survival rates. High-frequency Chinese herbs will be extracted from those beneficial prescriptions. Whereas, there are still large gaps in our understanding of TCM, such as what are the essential effective components in TCM, what are the key potential targets, and whether interplays exist between these herbal components. As a result, we performed network pharmacology analysis to explore the potential pharmacological mechanisms and targets of TCM in the treatment of GC.

## 2 Methods

### 2.1 Protocol and Guidance

This study adhered to the Preferred Reporting Items for Systematic Reviews and Meta-Analysis (PRISMA) guidelines. The protocol for this review was registered with PROSPERO (CRD42021252500).

### 2.2 Search Strategy

Three investigators independently searched several databases: PubMed, EMBASE, Cochrane, Chinese biological medicine (CBM), China Wanfang database, China National Knowledge Infrastructure databases (CNKI), Chinese Clinical Trial Registry (ChiCTR), ClinicalTrials.gov, and the World Health Organization International Clinical Trials Registry Platform from inception to September 26, 2021. Moreover, we also obtained the data sources from the abstracts and presentations recorded at some annual meetings, symposiums, or congresses such as ASCO, ESMO, ESGO, and so on, to ensure the relevant minutes were not overlooked. The complete search strategy was introduced in the **Supplementary Material**.

### 2.3 Selection Criteria

#### 2.3.1 Inclusion Criteria

Studies were included if they met the following criteria: if the people enrolled were GC patients (diagnosed based on histological or cytological results); if the subjects included had no contraindication to chemotherapy and no other systemic disease and were expected to live longer than 3 months; if TCM was in combination with chemotherapy for GC in the intervention group; if patients were treated with no TCM, or placebo, or conventional chemotherapy alone in the control group; if tumor response rate, QOL, AEs, serum tumor marker, and peripheral blood lymphocyte levels were reported (at least one of these outcomes); and if they were randomized controlled trials (RCTs).

#### 2.3.2 Exclusion Criteria

We excluded studies if they had not met our baseline requirements; if they employed the methods of acupuncture, massage, and other TCM treatment; if they did not offer the outcomes we needed; and if they were case reports, case series, observational studies, or other types of articles.

### 2.4 Study Selection and Data Extraction

Three independent investigators finished this part following the requirements of PRISMA according to the above inclusion and exclusion criteria. All disagreements about selection were discussed and resolved by all investigators. The data extraction included the author, year, country, research type, number of cases, age of patients, and the indicators for demonstrating outcomes. Tumor response was the primary outcome, including complete response (CR), partial response (PR), stable disease (SD), and progressive disease (PD). The CR rates combined with PR rates were equal to the ORR; the CR, PR, and SD rates were equal to the DCR. Quality of life (QOL) and the incidence of AEs were also the main outcomes. Immune function (peripheral blood lymphocyte levels), KPS score, and serum tumor marker were the secondary outcomes. Peripheral blood lymphocytes were evaluated by the levels of CD3^+^ T, CD4^+^ T, CD8^+^ T, and CD4^+^/CD8^+^ T ratio. Serum tumor markers were evaluated by CEA, AFP, and other common tumor markers.

### 2.5 Risk of Methodological Bias Assessment

Three researchers independently carry out the literature quality evaluation by applying the Cochrane evaluation handbook of RCTs (5.1.0), which includes the following characteristics covering randomization sequence generation (selection bias), allocation concealment (selection bias), blinding of participants and personal (performance bias), blinding of outcome assessment (detection bias), incomplete outcome data (attrition bias), selective reporting (reporting bias), and other bias. We used the Review Manager 5.3 software risk assessment tool to finish this part, and we decided through discussion or consultation with a third party when opinions were inconsistent.

### 2.6 Statistical Analysis

RevMan (version 5.3.3; The Cochrane Collaboration) and STATA (version 15; Stata Corporation, College Station, Texas, USA) were utilized to perform statistical analyses including pooling the data and producing the forest plots.

The combination of relative ratio (RR) and mean difference (MD) with a 95% confidence interval (CI) was used to assess outcomes and considered a *p*-value less than 0.05 to be statistically significant. The *I*² test was also used to assess the between-study heterogeneity. If the heterogeneity test is *p* ≥ 0.1 and *I* ^2^ ≤ 50%, it indicates that studies are homogenous between studies, and the fixed-effects model is used for combined analysis. Otherwise, we used a random‐effects model to pool effect size. *I*² regarded an estimated value applied to three fixed knots at 25%, 50%, and 75% as an indicator of mild, moderate, and high heterogeneity. Sensitivity analysis was also performed for examining the robustness of included trials to different aspects from methodological bias. In addition to this, publication bias was also estimated by an inverted funnel plot.

### 2.7 Acquisition of GC Disease Targets

In the NCBI (https://www.ncbi.nlm.nih.gov/), we selected the database GEO datasets. GMS mainly includes vulvar neoplasms (VN), vaginal cancer (VC), uterine cervix cancer (UCC), endometrial carcinoma (EC), OC, and fallopian tube carcinoma (FTC).VC and FTC were too few to have reliable disease samples in the GEOncbi database. Targets of VN, EC, and OC were mainly obtained, while the reliable disease samples of VC and FTC were too few to acquire. We searched for “vulvar neoplasm,” “uterine neoplasms,” and “ovarian neoplasms,” respectively. On the left, only series and expression profiling by an array of human tags are retained to narrow the screening range and obtain the corresponding samples of three tumor diseases (11–13). The platform file GPL571-17391 was selected, and the matrix file GSE63678 was standardized with Perl. The probe matrix was transformed into ID, and the whole gene matrix of VN was obtained. Platform files GPL10558-50081 and GPL887-20438 were then selected, respectively, and the matrix files GSE122697 and GSE12470 were standardized above. Finally, the whole gene matrices of EC and OC were acquired, followed by the differences in the GEO database analyzed for the three groups of gene data. The AveExpr of each gene in all samples was calculated using the R language, and the *p*-value was numerically corrected. Accordingly, the limma function package was used to construct a comparison matrix based on the ratio of the mean of the experimental group and the normal group, which was compared with the experimental design matrix. The eBayes function was used to make statistical inferences and analyze the difference. Finally, the corresponding gene of |log FC|>1, adj.*p*-val<0.05 was screened out.

### 2.8 Acquisition of Target in the Chinese Materia Medica Group

Traditional Chinese Medicine Systems Pharmacology Database and Analysis Platform (TCMSP) was used for obtaining the effective components of each TCM. The medicinal DL ≥0.18 condition played an important role in screening the effective components of TCM. Related targets were entered, and the corresponding targets of the selected active ingredients were derived. Perl has been used as an important tool for gene standardization, converting full names to genetic symbols.

### 2.9 Construction of a Regulatory Network of TCM

A variety of active ingredients and corresponding targets of TCM were retrieved from the TCMSP database. Perl was used to form the “component-target” database of the active ingredients and GC disease targets in **Section 2.7**. The intersection of the two was taken, and a Venn diagram was drawn.

### 2.10 Construction and Analysis of Protein (PPI) Interaction Network

The intersected genes were imported into the STRING database (https://string-db.org/), the species was limited to humans, and a score of >0.9 was set to construct the protein interaction network diagram of the TCM group-GC. The data were then saved as TSV files, and the information of Node1, Node2, and combined score are imported into Cytoscape V3.7.2. Finally, the proximity centrality and intermediary centrality of each node are calculated by using the CytoNCA function package and a reference degree value. Finally, the core gene targets with the highest significance in topology analysis were selected.

### 2.11 Gene Body Function and KEGG Pathway Enrichment Analysis

To clarify the role of target protein in gene function, gene ontology (GO) and Kyoto Encyclopedia of Genes and Genomes (KEGG) enrichment analysis should be performed for the intersection genes of TCM group-GC using the R language. First, the culsterProfiler package is imported, and the enrichGO function is used to import the human gene database through org.Hs.eg. and db function packages according to the intersection gene ID obtained previously. Under the conditions of “pvalueCutoff=0.05” and “qvalueCutoff=0.05,” the ID is converted into gene names and output to BP, CC, and MF groups for the GO enrichment analysis. Finally, the enrichPLOT and GGploT2 function packages are used to draw visual diagrams. Secondly, the KEGG enrichment analysis is carried out using the enrichKEGG function, and then the pathway—gene files and node files obtained by Perl are imported into CytoscapeV3.7.2 to build a KEGG network. Based on the above enrichment analysis results, we can predict the action mechanism of GC treated by various TCM.

## 3 Results

### 3.1 Identification and Selection

**Figure 1:**
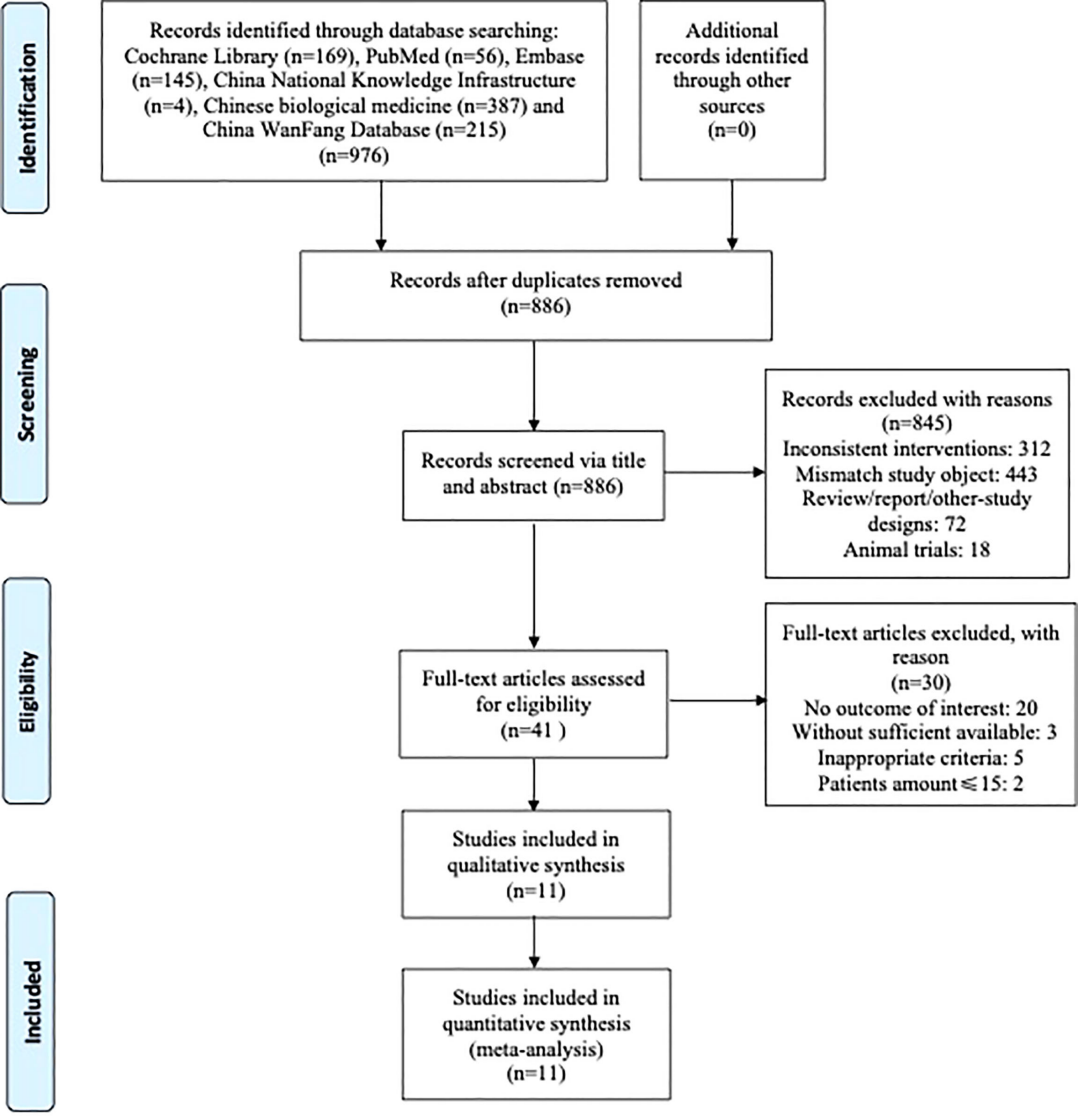
Flow diagram indicating the process of selecting articles for meta-analysis and the steps of network pharmacology analysis.

As shown in **Figure 1**, 976 articles from online databases and other manual sources were initially obtained in the literature search, of which 90 publications were excluded for duplications. Also, after browsing titles and abstracts, 845 irrelevant articles were excluded. We continued to review the remaining 41 potentially eligible articles by full text based on the inclusion and exclusion criteria; 11 RCTs (14–24) met the inclusion criteria and were left for further analysis at last.

### 3.2 Characteristics of Included Studies and Patients

The main characteristics of the 11 included studies are shown in **Table 1**. All trials were RCTs and conducted in China. The publication years ranged from 2001 to 2020, and there were a total of 863 women enrolled for the analysis, with a total of 465 in the intervention group and 398 in the control group. All the intervention groups used TCM plus chemotherapy, while all the control groups used chemotherapy. Four trials reported tumor responses, and four trials reported QOL data. Seven trials reported the peripheral blood lymphocyte levels, and 3 trials mentioned the results of AEs. No trials reported the results of tumor markers, and 2 trials mentioned incomplete KPS results that could not be extracted for meta-analysis.

**Table 1:** Characteristics of studies included in this meta-analysis.

Author (year)	Research type	Number of cases		Age		Intervention measurement	
		Combination therapy	Chemotherapy	Combination therapy	Chemotherapy	Combination therapy	Chemotherapy
Liu (2001)(19)	OC	90	30	15–77	17–22	Chemotherapy + Yiliu Fang	DDP, 5-FU, CTX, ADM,VP-16, PTX
Gong (2018)(18)	UCC	32	32	47.9 ± 2.4	48.2 ± 2.5	Chemotherapy + Fuzheng drink I	PTX, DDP
Zhang (2019)(23)	OC	45	45	53.5 ± 6.8	52.7 ± 8.2	Chemotherapy + Yiqi anticancer prescription	PTX, DDP
Nan (2015)(20)	UCC	30	30	48.8 ± 6.1	48.7 ± 6.3	Chemotherapy + Bazhen decoction	Irinotecan, DDP
Song (2020)(21)	OC	68	68	66. 79 ± 4. 15	65.46 ± 4.21	Chemotherapy + Fuzheng Jiedu decoction	PTX, DDP
Zhong (2010)(24)	GC	26	22	48.57 ± 6.83		Chemotherapy + Jianpi Bushen decoction	5-FU, PTX, DDP
Gao (2020)(16)	UCC	46	46	42.15 ± 2.91	42.47 ± 2.99	Chemotherapy + Shugan Huayu Jiedu	Docetaxel, oxaliplatin
Yu (2018)(22)	GC	30	30	65.00 ± 10.84	64.03 ± 10.14	Chemotherapy + modified Qi-tonifying and blood-activating prescription	Conventional chemotherapy
Zhang (2006)(14)	GC	25	25	51.24 ± 8.97	50.64 ± 1028	Chemotherapy + TCM	5-FU, PTX, DDP
Huang (2017)(18)	OC	42	42	39.9 ± 16.0	39.7 ± 15.4	Chemotherapy + Guizhi Fuling pill	PTX, CBP
Chan (2011)(15)	OC	31	28	52.9	51.5	Chemotherapy + TCM	Conventional chemotherapy

OC, ovarian cancer; DDP, cisplatin; 5-FU, 5-fluorouracil; CTX, cyclophosphamide; ADM, adriamycin; VP-16, etoposide; PTX, paclitaxel; UCC, uterine cervix cancer, GC, gynecological cancer; TCM, traditional Chinese medicine; CBP, carboplatin.

### 3.3 Assessment of Methodological Bias

**Figure 2:**
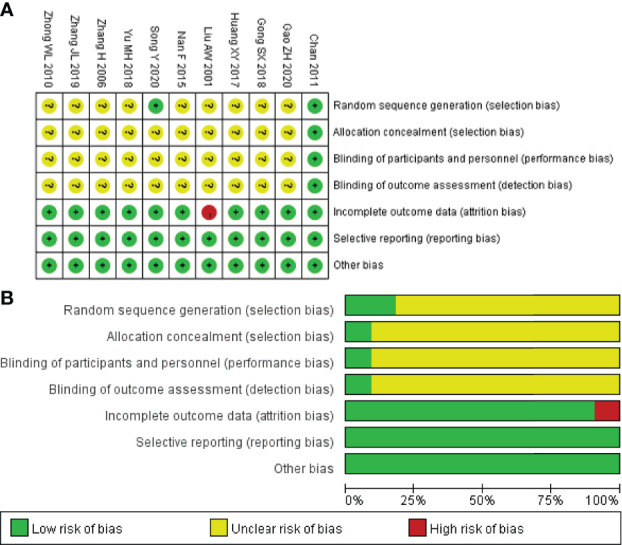
Risk of bias summary **(A)** and diagram **(B)**.

All of the 11 studies referred to randomization. Only 2 trials (15, 21) used a random number table that generated a random sequence, while the other did not mention the method. Only Chan (15) performed the allocation concealment by block randomization, while other studies did not report this method. Also, only one study (15) mentioned double-blinding, while the rest of the studies failed to report the blinding of the investigators, patients, and outcome assessors. Incomplete outcomes existed in Liu (19). In addition, all trials were supposed to have a low risk of other bias and did not have selective reporting. The quality assessment of the included studies is summarized in **Figure 2**.

### 3.4 Efficacy

#### 3.4.1 Tumor Response

**Figure 3:**
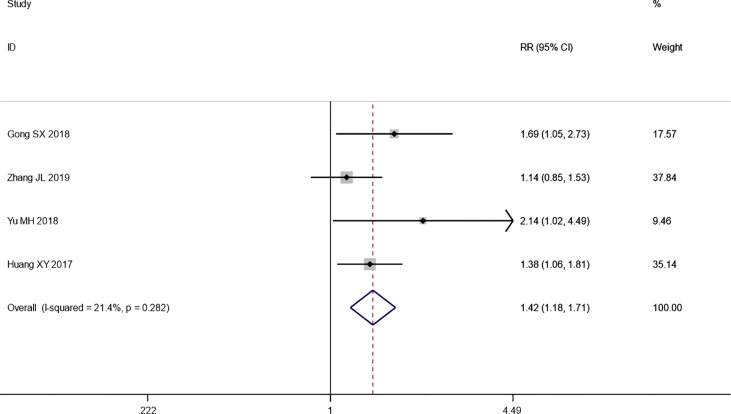
Forest plot of the meta-analysis of the objective response rate (ORR) between the intervention group and the control group.

**Figure 4:**
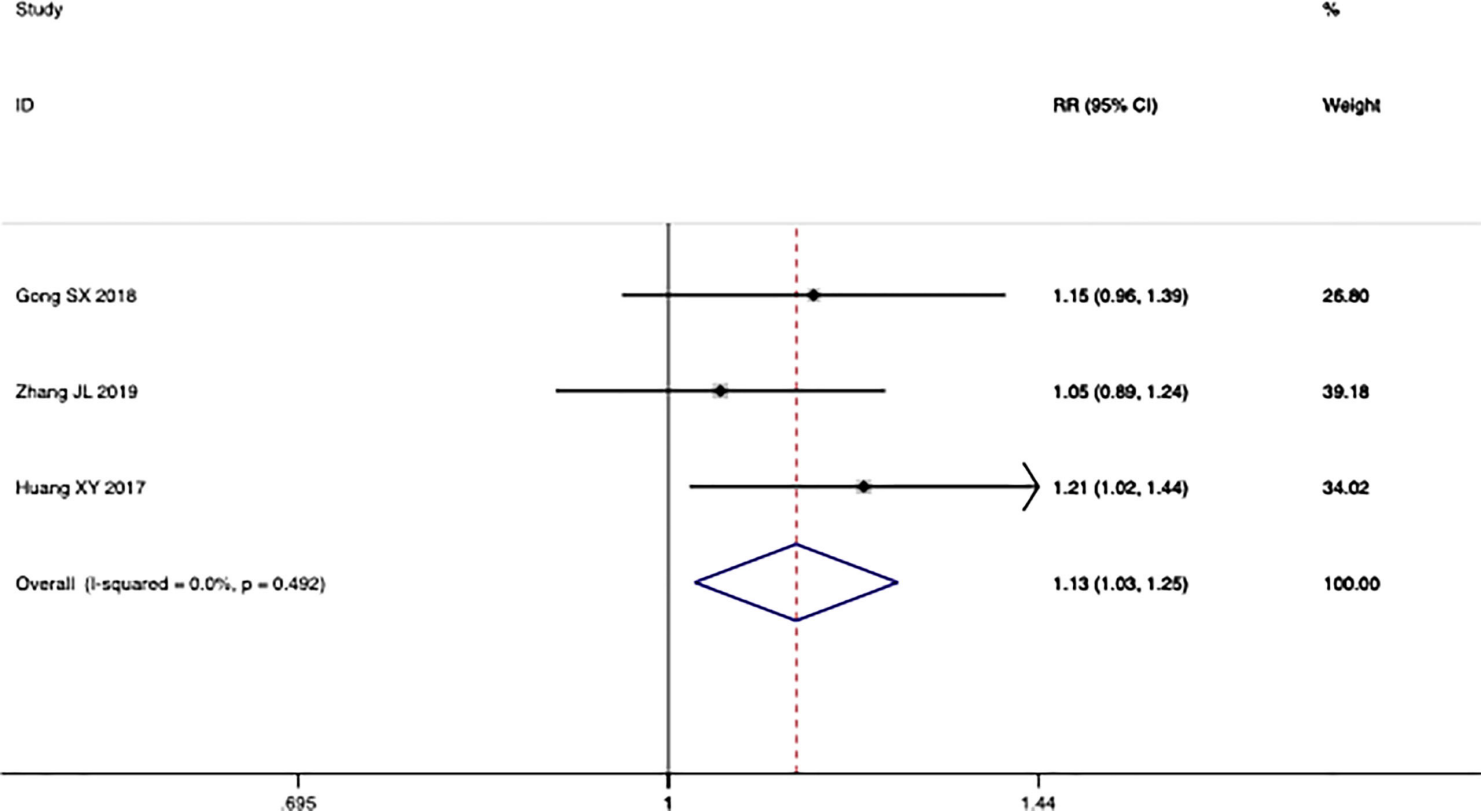
Forest plot of the meta-analysis of the disease control rate (DCR) between the intervention group and the control group.

**Figure 5:**
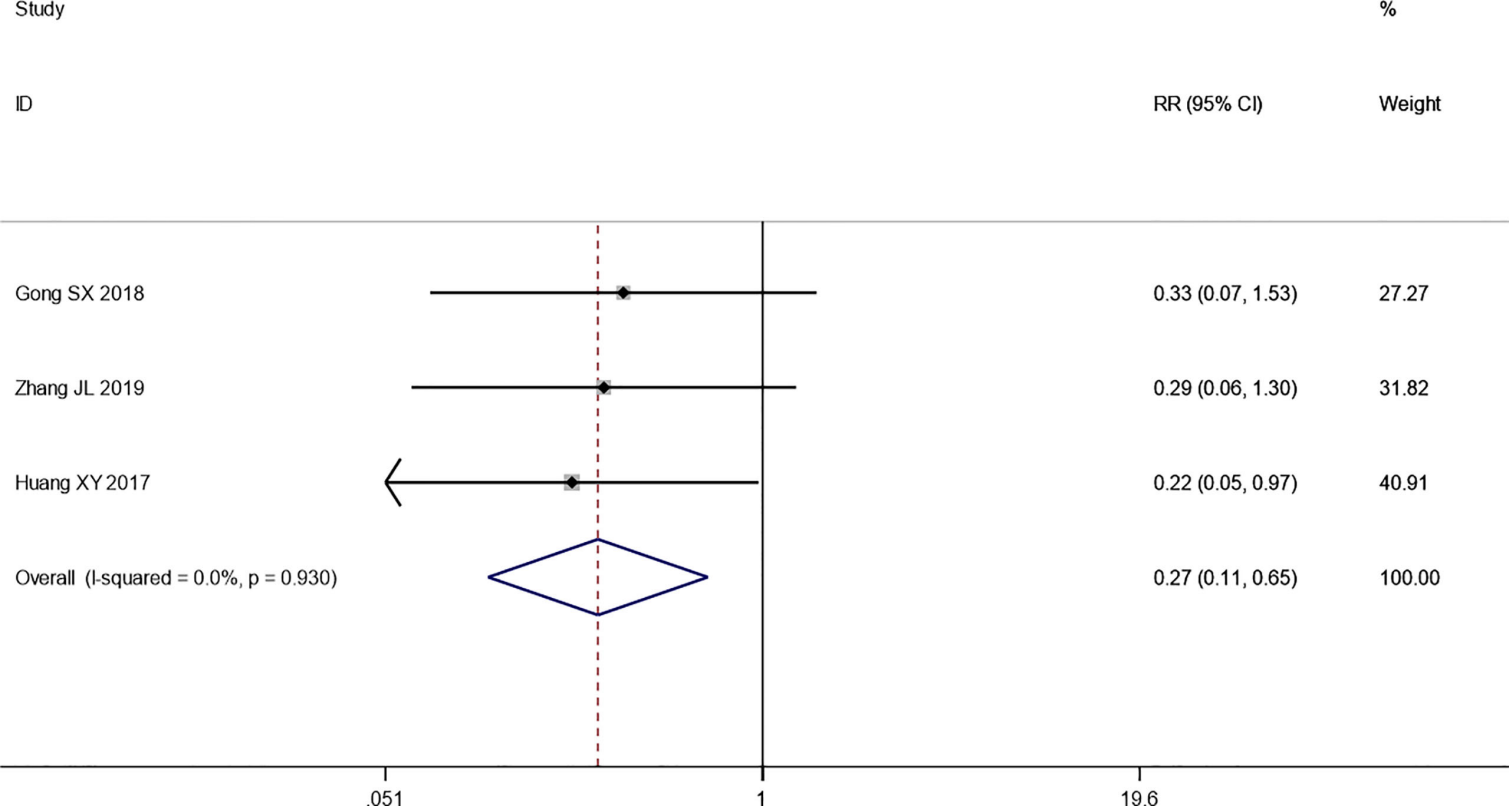
Forest plot of the meta-analysis of progressive disease (PD) between the intervention group and the control group.

Four trials reported the ORR following WHO or RECIST guidelines. The pooled results show that the ORR of the combination therapy group was significantly higher than that of the chemotherapy-alone group (RR: 1.42; 95% CI: 1.18–1.71; *I* ^2^ = 21.4%; *p* *=* 0.000) (**Figure 3**). Moreover, three trials reported the result of SD and PD. The pooled results show that DCR of combination therapy group was also higher than control group (RR: 1.13; 95% CI: 1.03–1.25; *p* *=* 0.110; *I* ^2^ = 0.0%, *p* *=* *0.492*) (**Figure 4**). While the pooled results show that PD of combination therapy group was significantly lower than that in chemotherapy-alone group (RR: 0.27, 95% CI: 0.11–0.65, *p* = 0.003; *I* ^2^ = 0.0%, *p* = 0.930”) (**Figure 5**).

#### 3.4.2 Quality of Life

**Figure 6:**
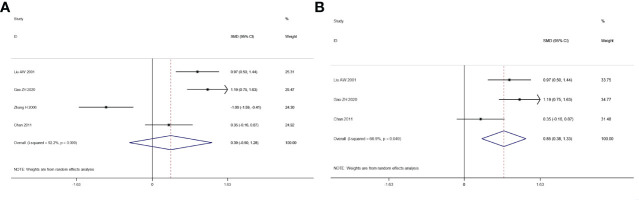
Forest plot of the meta-analysis of quality of life (QOL) before sensitivity analysis **(A)** and after sensitivity analysis **(B)** between the intervention group and the control group.

We explored the quality of life of patients who suffered from integrated traditional Chinese medicine plus chemotherapy and chemotherapy alone. The result of the heterogeneity test is: *I* ^2^ = 92.2%, *p* *=* 0.000 (**Figure 6A**). Since the sensitivity analysis showed that Yu’s research had a great influence on the results, we eliminated the literature and then summarized the results again. After excluding the study, the heterogeneity had been significantly reduced (*I* ^2^ = 66.9%, *p* *=* 0.049), and a meta-analysis was conducted through a random-effects model. The pooled results show that the quality of life of the combination therapy group is significantly better than that of the chemotherapy alone group (SMD: 0.85, 95% CI: 0.38–1.33, *p* *=* 0.005) (**Figure 6B**).

#### 3.4.3 Peripheral Blood Lymphocyte Levels

**Figure 7:**
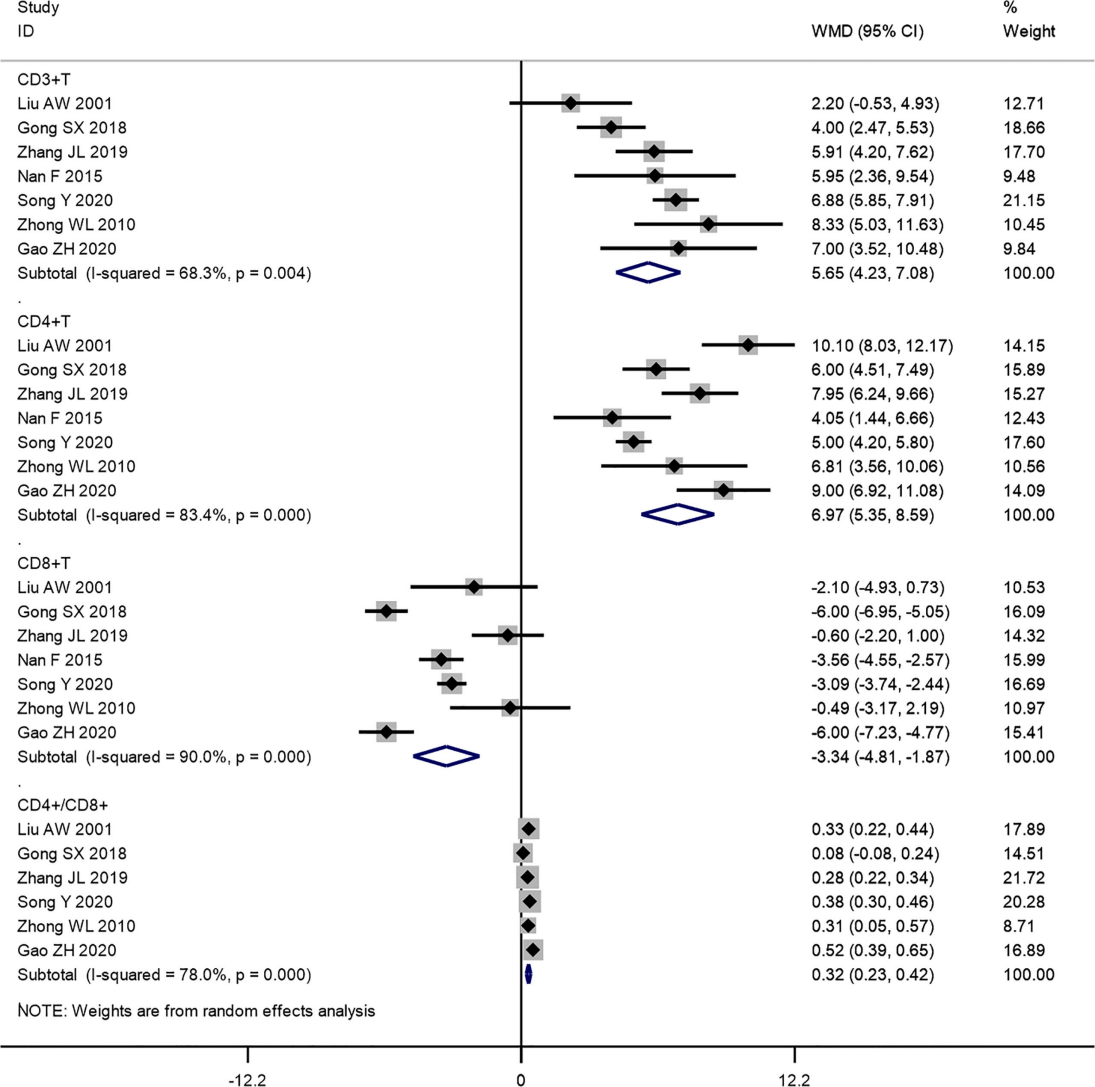
Forest plot of the meta-analysis of immune function indicators including CD3^+^ T, CD4^+^ T, CD8^+^ T, and CD4^+^/CD8^+^ T cells between the intervention group and the control group.

Additionally, we analyzed the levels of CD3^+^ T, CD4^+^ T, CD8^+^ T, and CD4^+^/CD8^+^ T ratio of patients suffering from integrated traditional Chinese medicine plus chemotherapy and chemotherapy alone. The pooled results show that the levels of CD3^+^ T (WMD: 5.65, 95% CI: 4.23–7.08, *p* = 0.000; *I* ^2^ = 68.3%, *p* = 0.004), CD4^+^ T (WMD: 6.97, 95% CI: 5.35–8.59, *p* = 0.000; *I* ^2^ = 83.4%, *p* = 0.000), and CD4^+^/CD8^+^ T ratio (WMD: 0.32, 95% CI: 0.23–0.42, *p* = 0.000; *I* ^2^ = 78.0%, *p* = 0.000) of the combination therapy group were significantly higher than those in the chemotherapy-alone group. While the level of CD8^+^ T cells of the combination therapy group didn’t show an obvious increase (WMD: -3.34, 95% CI: -4.81~ -1.87, p = 0.000; *I* ^2^ = 90.0%, p = 0.000) (**Figure 7**).

#### 3.4.4 Safety

**Figure 8:**
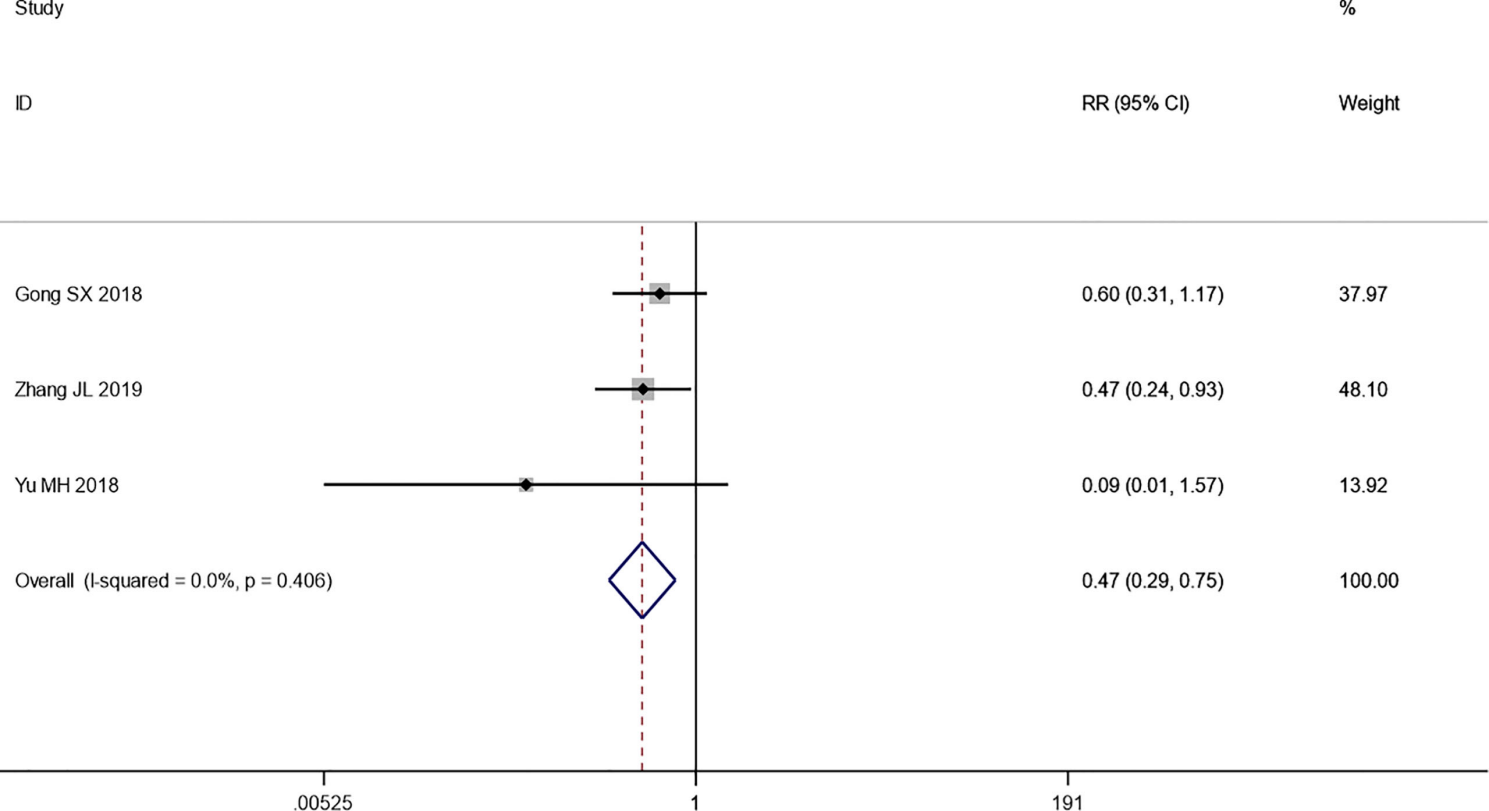
Forest plot of the meta-analysis of AEs between the intervention group and the control group.

Lastly, we pooled the incidence of AEs including gastrointestinal reactions, hepatic and renal dysfunction, fatigue, alopecia, stomatitis, and myelosuppression. However, we could only compare these two treatments by the number of AEs because the data did not provide a classification of them. A meta-analysis was conducted through a fixed-effects model, and the pooled results showed that the incidence of AEs in the combination therapy group was significantly lower than that in the chemotherapy-alone group (RR: 0.47, 95% CI: 0.29–0.75, *p* = 0.002) (**Figure 8**).

#### 3.4.5 Sensitivity Analysis

Sensitivity analysis eliminates each included study one by one and performs a summary analysis on the remaining studies to assess whether a single included study has an excessive impact on the results of the entire meta-analysis. The result of the sensitivity analysis is shown in **Supplementary Figures S1–S9**. The results showed that almost no studies had an excessive impact on the results of the meta-analysis, indicating that the results of the remaining studies are stable and reliable.

#### 3.4.6 Publication Bias

**Figure 9:**
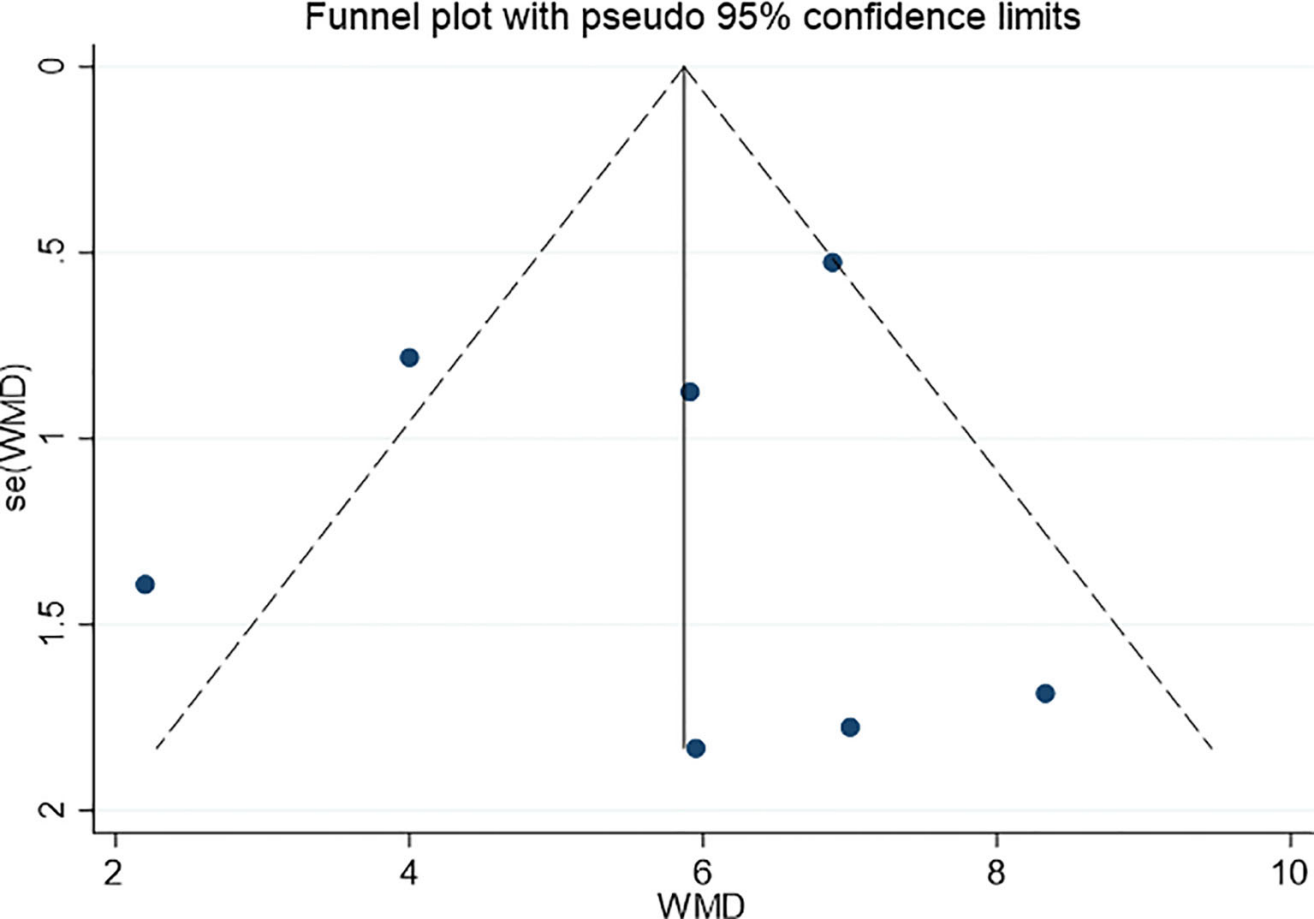
Funnel plot for evaluating the publication bias of this meta-analysis.

The funnel plot is shown in **Figure 9**. It can be seen that the funnel plot is not symmetrical, indicating that there may be publication bias in this study.

#### 3.4.7 Effective Herbs Extraction

To discern the commonality of the TCM formulae for combined chemotherapy in the included studies, we analyzed the occurrence frequency and compatibility of the compounds in those Chinese herbal prescriptions.

**Figure 10:**
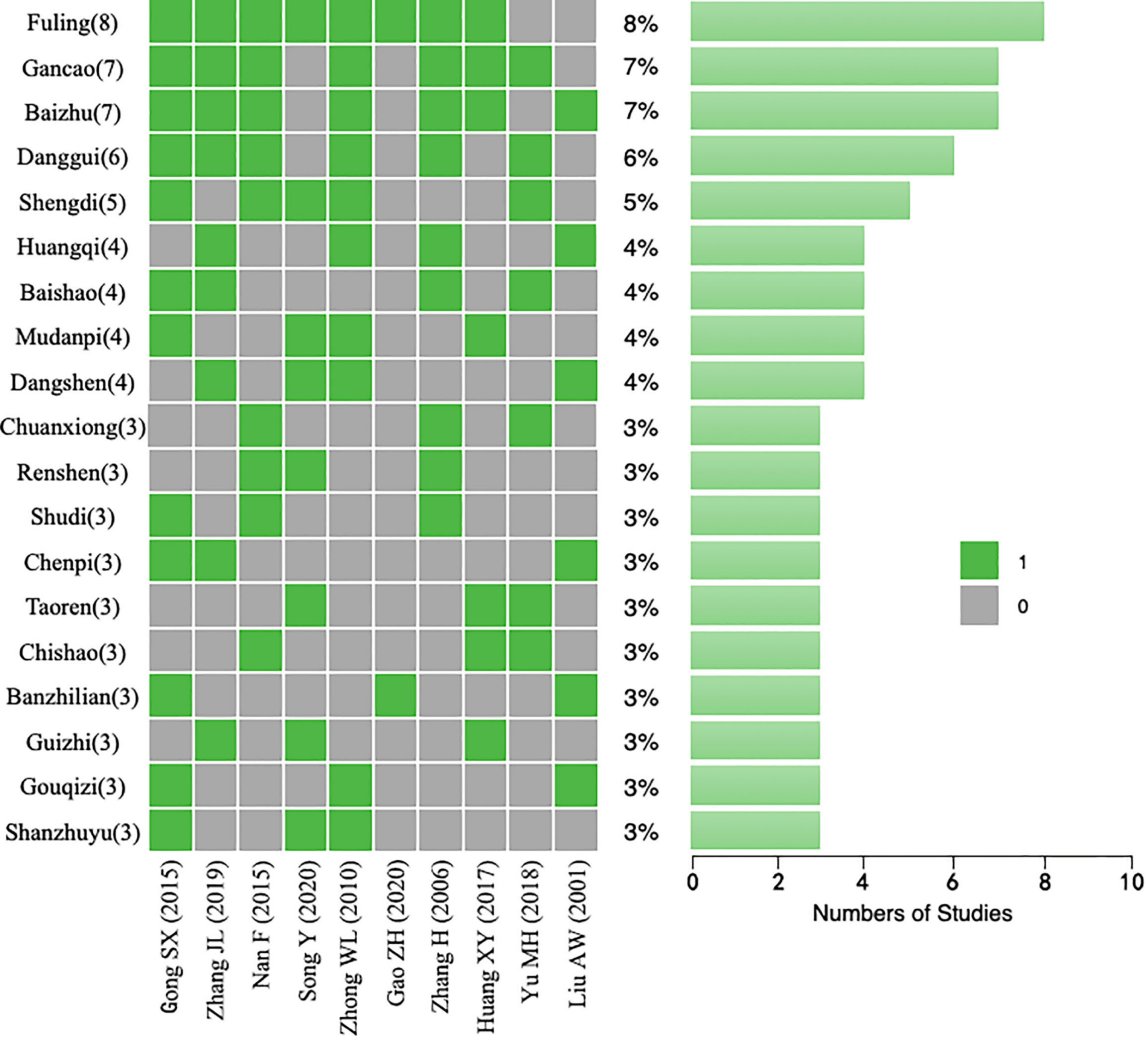
Traditional Chinese medicine (TCM) herbs that appeared in each study.

We analyzed the characteristics of the 10 different TCM prescriptions mentioned in the included trials. Ordered according to their frequency of use, the TCM whose counts were more than three are listed in **Figure 10** and **Table 2**, namely Fuling, Gancao, Baizhu, Danggui, Shengdi, Huangqi, Baishao, Mudanpi, Dangshen, Chuanxiong, Renshen, Shudi, Chenpi, Taoren, Chishao, Banzhilian, Guizhi, Gouqiz, and Shanzhuyu.

**Table 2:** The high-frequency Chinese herbs in each study.

Chinese name	Pharmaceutical name	Counts	Frequency 1 (counts/total herb counts)	Frequency 2 (counts/study numbers)
Fuling	Poria cocos	8	7.27%	80.00%
Gancao	Liquorice	7	6.36%	70.00%
Baizhu	Macrocephala	7	6.36%	70.00%
Danggui	Angelica sinensis	6	5.45%	60.00%
Shengdi	Radix Rehmannia	5	4.55%	50.00%
Huangqi	Astragalus	4	3.64%	40.00%
Baishao	Radix paeoniae alba	4	3.64%	40.00%
Mudanpi	Cortex moutan	4	3.64%	40.00%
Dangshen	Codonopsis pilosula	4	3.64%	40.00%
Chuanxiong	Ligusticum chuanxiong	3	2.73%	30.00%
Renshen	Ginseng	3	2.73%	30.00%
Shudi	Radix rehmannia praeparata	3	2.73%	30.00%
Chenpi	Pericappium citrus reticulata	3	2.73%	30.00%
Taoren	Semen persicae	3	2.73%	30.00%
Chishao	Radix paeoniae rubra	3	2.73%	30.00%
Banzhilian	Scutellaria barbata	3	2.73%	30.00%
Guizhi	Ranmulus cinnamomi	3	2.73%	30.00%
Gouqizi	Fructus lycii	3	2.73%	30.00%
Shanzhuyu	Cornus officinalis	3	2.73%	30.00%

In addition, 4 of these studies (40%) used a combination of Dangshen/Renshen, Gancao, Baizhu, and Fuling, which is the most representative prescription for nourishing qi and is known as Sijunzi decoction. Based on these findings, it can be inferred that in the treatment of GC by TCM + chemotherapy, using basic qi tonic TCM to invigorate qi and strengthen the spleen can be an effective method, which was also found in some experiments. A new effective prescription was composed of these 19 herbs for subsequent network pharmacology analysis.

#### 3.4.8 Screening of Effective Components in TCM Group

Through the above meta-analysis, 19 kinds of TCM (Fuling, Gancao, Baizhu, Danggui, Shengdi, Huangqi, Baishao, Mudanpi, Dangshen, Chuanxiong, Renshen, Shudi, Chenpi, Taoren, Chishao, Banzhilian, Guizhi, Gouqiz, Shanzhuyu) have good effects on GC treatment. Due to the large number of Chinese medicines, we took Counts4 as the screening standard, and took the first 9 Chinese medicines as the TCM group.

Using the TCMSP database, the TCM group was entered into the database, and the obtained ingredients were screened according to oral bioavailability (OB) of ≥30% and drug like (DL) of ≥0.18. Through searching the TCMSP database, there is no effective ingredient in Shengdi that meets ADME standards. Therefore, the remaining 8 TCM in the TCM group are used to screen the effective ingredients. Finally, there were 6 active components in Fuling, 88 active components in Gancao, 4 active components in Baizhu, 2 active components in Danggui, 16 active components in Huangqi, 8 active components in Baishao, 3 active components in Mudanpi, and 12 active components in Dangshen. After weight removal, there were 120 active components in the TCM group.

#### 3.4.9 Screening of Highly Correlated Targets for GC Disease

The GEO database difference analysis was performed on the whole gene matrix of VN, EC, and OC obtained in **Section 2.7**. The genes with a high correlation with GC in |log FC|>1 adj.*p*-val<0.05 were screened by the method in **Section 2.7**.** A** total of 626 high correlation targets in VN, 3,884 high correlation targets in EC, and 5,665 high correlation targets in OC were obtained. As there were few highly correlated targets in VN, we expanded the screening conditions and screened |log FC|>0.5 *p*-val<0.05 as a highly correlated gene of VN. A total of 2,144 highly correlated targets in VN were obtained. After deleting the overlapping targets in the three groups of data, 8,949 high correlation targets of GC disease were obtained.

#### 3.4.10 Construction of a Regulatory Network for the TCM Group

**Figure 11:**
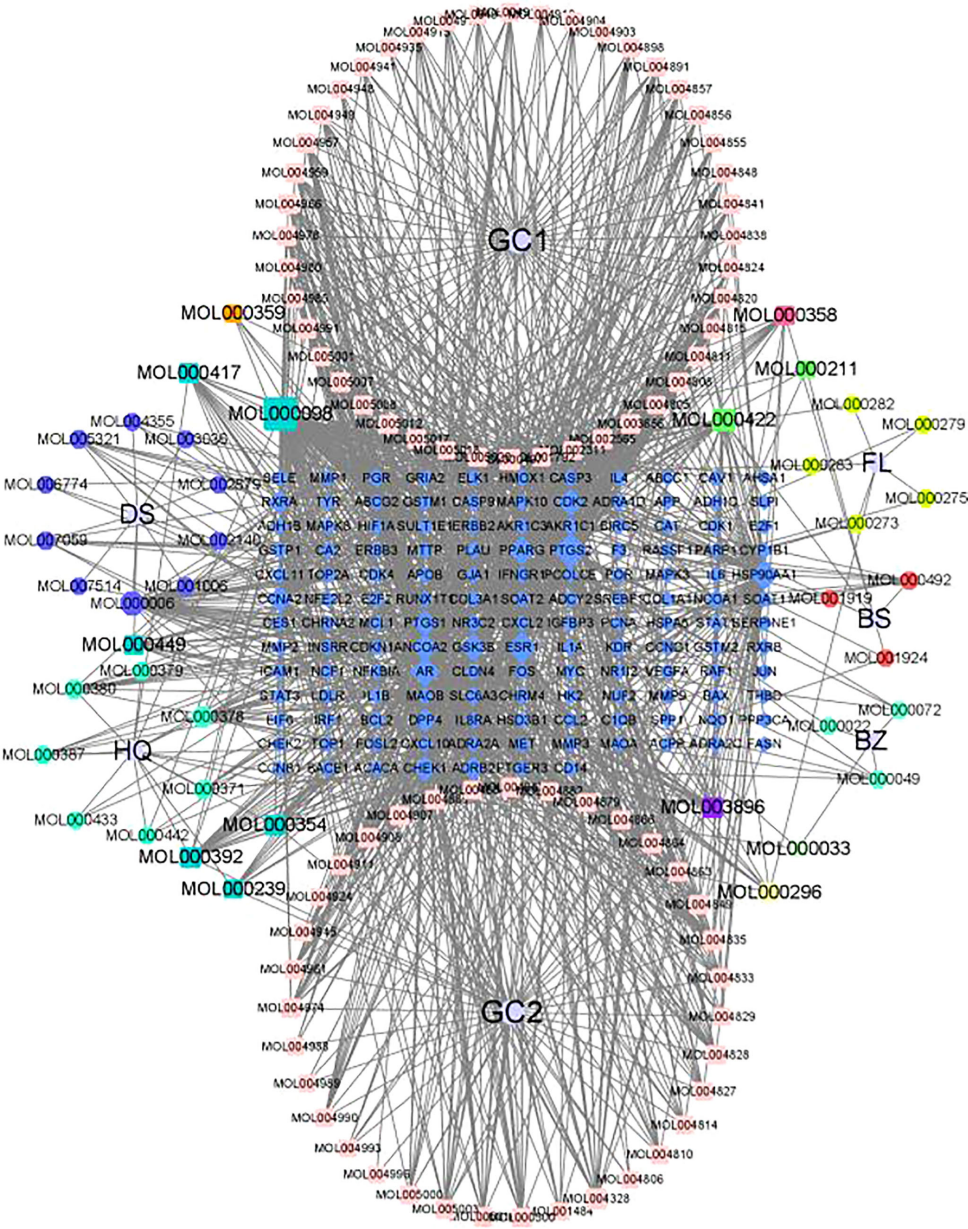
TCM group regulatory network.

In total, 120 active components were retrieved from the TCMSP database, and 225 directly corresponding targets were obtained after weight removal. A total of 8,949 highly correlated targets of GC disease were retrieved from the GEO and GeneCards databases, including 139 drug-disease common targets. After standardizing the gene, the full name of the gene is transformed into the gene symbol. Finally, Cytospace3.7.2 software was used to construct the regulation network of the TCM group (**Figure 11**).

#### 3.4.11 Construction and Analysis of PPI Network of GC Disease Target in TCM Group

**Figure 12:**
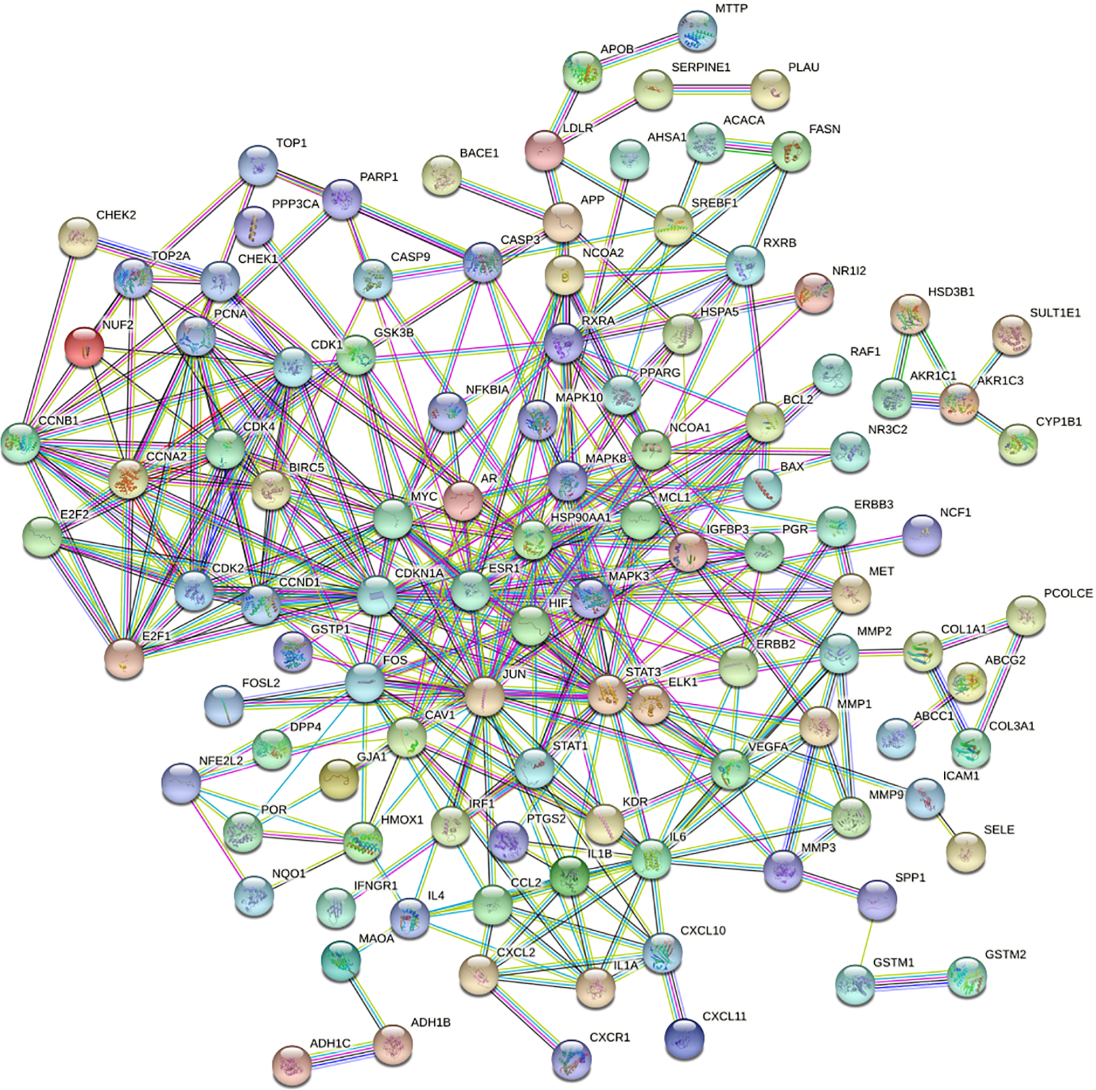
Protein interaction network.

The intersection genes obtained in **Section 3.4.10** were imported into the STRING database, and the species was defined as human. A score of >0.9 was set to hide the disconnected nodes, and the interaction network between the TCM group and GC disease target proteins was constructed, which is illustrated in **Figure 12**.

**Figure 13:**
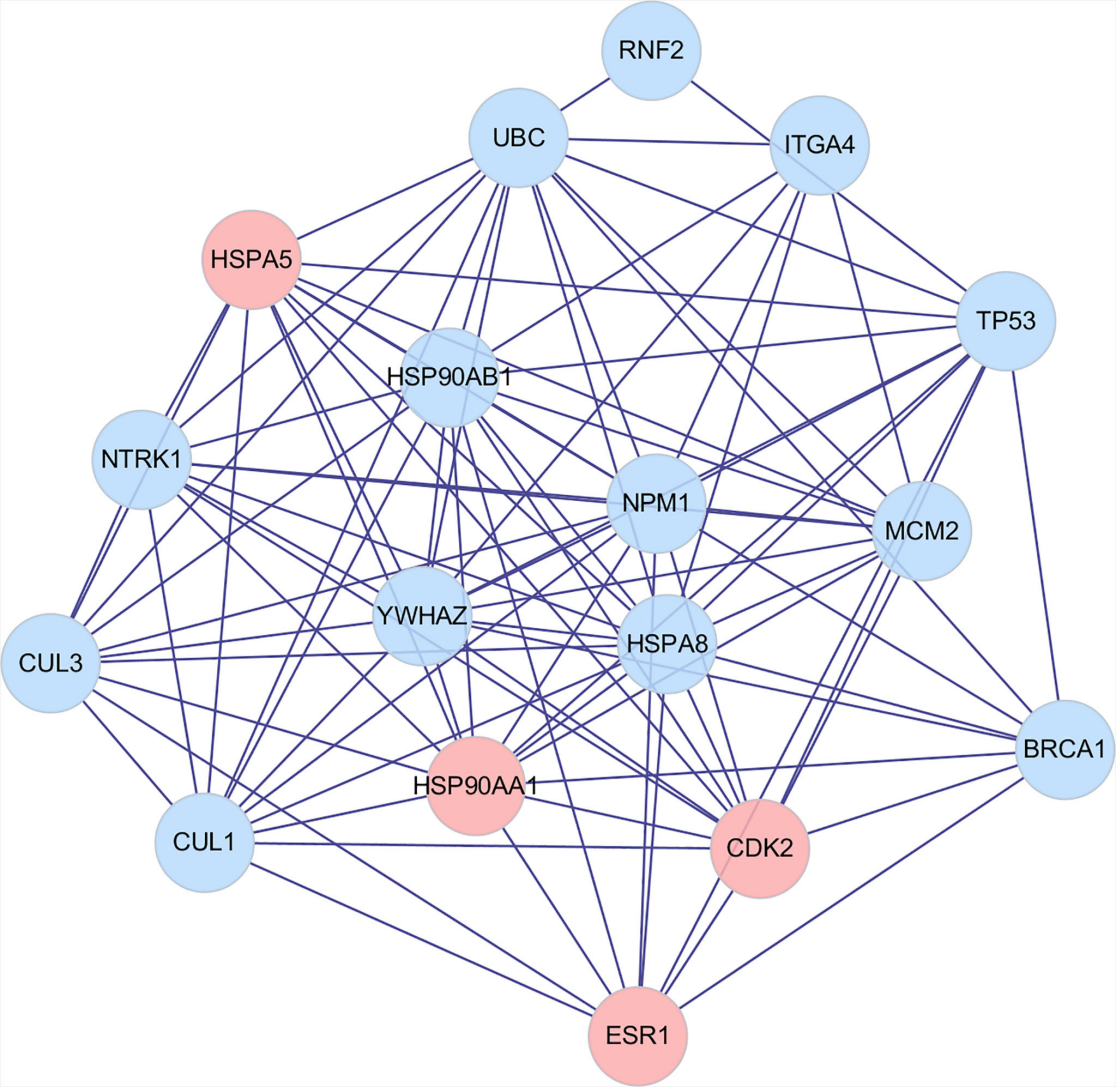
PPI core network.

Topology analysis of the protein–protein interaction (PPI) network was then required. The data in **Figure 13** were saved as a TSV file, and the information for Node1, Node2, and the combined score in the file was imported into the Cytoscape V3.7.2 software. Finally, BisoGenet and CytoNCA in Cytoscape software were used. Referring to the degree value, the “betweenness centrality (BC)” and “closeness centrality” of all nodes were calculated. The top genes of DC and BC were sequentially screened, and the protein interaction core of the TCM group was finally determined (**Figure 13**). The topology parameter analysis is shown in **Table 3**. 

**Table 3:** Analysis of topology parameters.

Number	Gene	Degree	BC	CC
1	NPM1	14	9.15	0.89
2	HSPA8	14	9.15	0.89
3	YWHAZ	14	7.18	0.89
4	HSP90AB1	14	6.97	0.89
5	UBC	13	21.78	0.84
6	HSP90AA1	13	4.79	0.84
7	CDK2	12	3.73	0.80
8	HSPA5	12	2.19	0.80
9	TP53	11	13.81	0.76
10	NTRK1	11	1.22	0.76

According to the results of topological parameter analysis, genes NPM1 and HSPA8 were the most critical genes in GC. Through the PPI core network, HSP90AA1, HSPA5, CDK2, and ESR1 were found to be the core genes directly associated with the effective components of the TCM group, among which HSP90AA1 ranked the highest.

#### 3.4.12 Functional Enrichment Analysis of Gene Body (GO)

An enrichment analysis was carried out for 139 intersection genes of the screened TCM group and GC disease targets, and the analysis results of biological process (BP), cell composition (CC), and molecular function (MF) involved in this core target could be obtained in **Supplementary Figures S10–S12**.

From the analysis of **Supplementary Figure S10**, the core target of metal ions in BP responses to the significance of the highest, followed by oxidation reaction, cell response to chemical stress, steroids, drug reactions, radiation reactions. Besides, the reaction of lipopolysaccharide and active oxygen reaction control have positive influence on nutrient levels and oxidative stress reaction. In terms of CC, the above core targets were most significant in membrane raft, plasma membrane microregion, and membrane region, followed by transcriptional regulatory complex, endoplasmic management cavity, RNA polymerase II transcriptional regulatory complex, organelle outer membrane, and other parts. In terms of MF, DNA-binding transcription factor binding was the most significant, followed by RNA polymerase II-specific DNA-binding transcription factor binding, amide binding, polypeptide binding, and cytokine receptor binding. Therefore, the TCM group may focus on the above biological functions to participate in the treatment of GC diseases.

#### 3.4.13 Enrichment Analysis of KEGG Pathway

**Figure 14:**
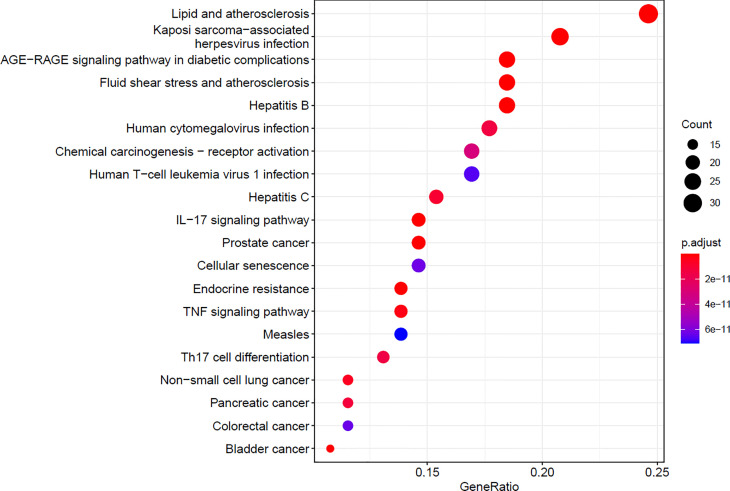
KEGG enrichment analysis.

Whether the Chinese traditional medicine group—the intersection of GC disease gene KEGG enrichment analysis, gets the core targets in lipid and atherosclerosis, Kaposi’s sarcoma-associated herpesvirus infections, hepatitis B, diabetes complications of AGE-RAGE signaling pathways, fluid shear stress and atherosclerosis, and the human cytomegalovirus pathway is salient. It indicates that the components in the TCM group may achieve the purpose of curing diseases by acting on the above signal pathways (**Figure 14**).

**Figure 15:**
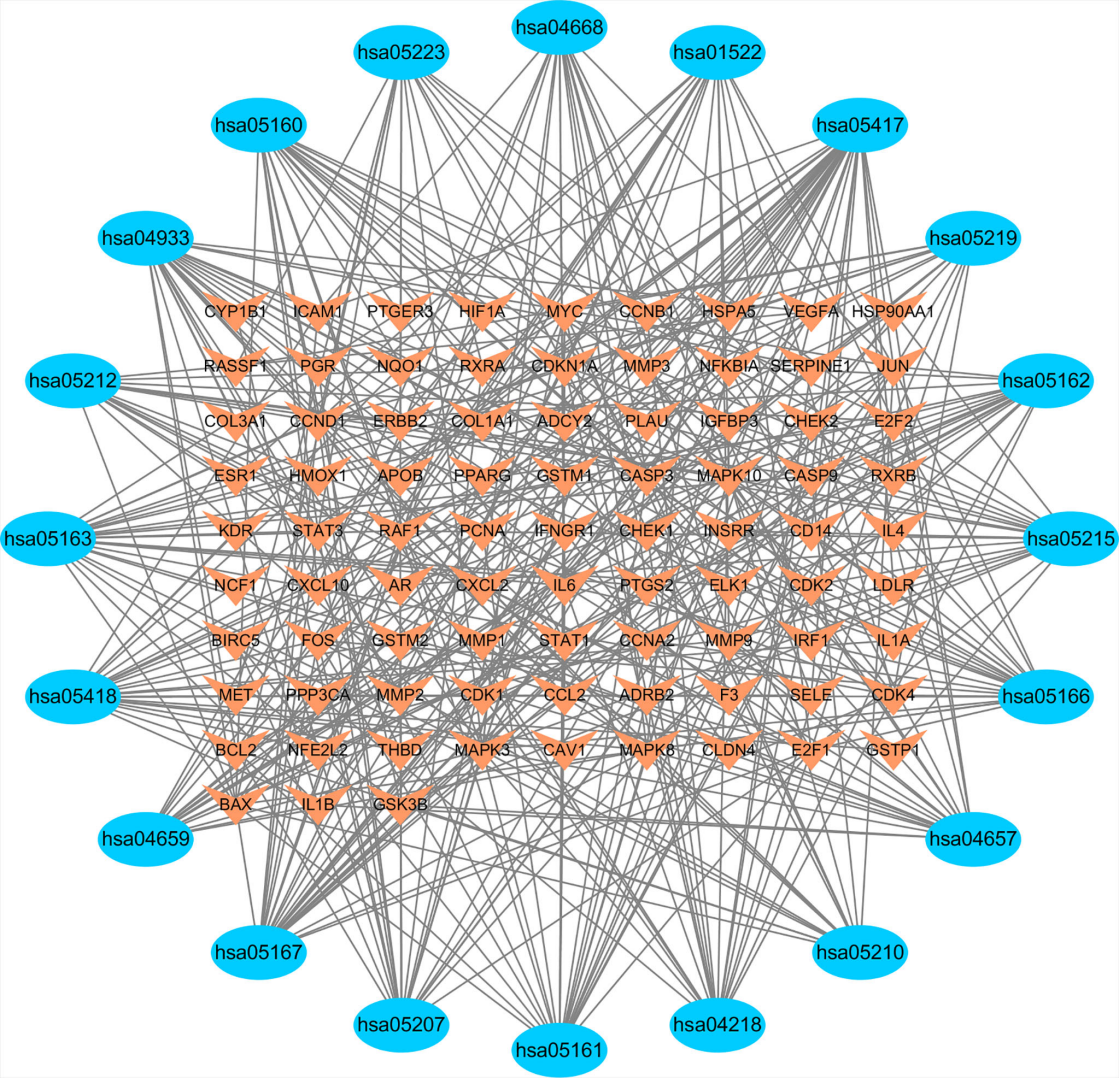
KEGG regulatory network.

Accordingly, the KEGG network is constructed, with the outer circle as the related pathway and the inner circle as the core gene, whose graph size is proportional to the number of associated pathways (**Figure 15**).

## 4 Discussion

Based on previous studies, it was demonstrated that TCM plays an important role in the treatment of GC, so we conducted a systematic review and meta-analysis integrated with network pharmacology analysis to compare the efficacy of TCM plus chemotherapy with chemotherapy alone, and to identify the potential pharmacological mechanisms and targets of TCM.

As far as our knowledge is concerned, this is the first article that integrated meta-analysis and network pharmacology to evaluate the efficacy and potential pharmacological mechanisms of TCM in patients with GC. Previously, Ge et al. (10) provided a comprehensive meta-analysis for people with OC, but they only investigated the efficacy of TCM concerning survival, QOL, and immune function without assessing its safety. Coincidentally, network pharmacology conducted by Sun et al. (25) also paid attention to the potential targets of TCM for OC patients. However, in our study, which included 11 RCTs consisting of 863 GC patients, it was shown that TCM plus chemotherapy can provide more durable disease control and improve GC patients’ QOL without substantially increasing AEs compared with chemotherapy alone, and the major ingredients and major targets involved in the effective treatments were also illustrated.

Based on the guidelines standardized by NCCN, ESMO, and ASCO, the treatment options for patients encompass surgery, chemotherapy, radiotherapy, biological therapy, targeted therapy, immunotherapy, or an integration of these options. Among them, chemotherapy has been successfully and commonly utilized in the treatment of GC. Platinum derivatives, paclitaxel, and antimetabolites, all alkylating drugs, are the most commonly used antitumor agents, whose mechanisms are based on the damage of genomic synthesis and repair mechanisms in cancer cells (26). It will cause extensive DNA damage that exceeds the cellular DNA repair capacity and ultimately leads to apoptotic cancer cell death. These drugs are usually nonselective to cancer cells, and consequently, they induce concurrent damage to healthy tissues and organs. Following chemotherapy, women will be at an increased risk of experiencing AEs, and the next course of treatment is likely to be delayed or terminated due to a high degree of drug toxicity and poor results from the evaluation of its efficacy. Recent studies have also found it is difficult to improve its therapeutic effects, and the response of patients is being reduced in the wake of each subsequent line of therapy (27, 28).

TCM occupies a very important position in the treatment of cancers in China. It has been reported to enhance cytotoxic effects and reduce side effects of chemotherapeutic agents. A bulk of studies have proved that TCM has a unique advantage in the treatment of GC. Research by Supoken et al. has shown that TCM can enhance the ability of the body’s immunity and resistance to tumors and can relieve chemotherapy-induced immunosuppression. Wu et al. (7) found that TCM could prolong the chemotherapy interval and decrease the incidence of AEs. It was also reported that TCM could effectively alleviate the AEs of chemotherapy and improve QOL (29). The results of our study were also consistent with them, but a few aspects still need to be considered when interpreting our findings.

Firstly, moderate to high heterogeneity was observed across all included trials, which may result from the types of TCM, tumor type, pathogenic type, cancer histotype, and line of treatment. We used sensitivity analysis to detect the article that affected the result and obtained a meaningful result by removing it. As for the newly achieved drug composition from those 11 RCTs, whether it could achieve certain therapeutics in the clinic still needs to be investigated. In our study, none of the included trials completely reported the overall survival (OS) or progression-free survival (PFS); they only illustrated the curative effects of factors including tumor responses, QOL, peripheral blood lymphocyte levels, and AEs in treating GC. Thus, more newly offered evidence needs to be stated to confirm whether TCM plus chemotherapy could prolong the long-term survival rates of GC patients. Among those trials, lacking the data on the applications of injection-based, pill, and external administration of TCM, the included formulas were all oral administration. Also, almost all TCM were used after the chemotherapy cycle ended. The utilization of TCM before or during the chemotherapy cycle still needs more data to support. More studies to determine the best dosage form and use time of TCM are needed in the future.

Myelosuppression, hepatic and renal dysfunction, and gastrointestinal reactions were common chemotherapy-related side effects (30, 31), which could reflect patients’ tolerance to chemotherapy. Dose modification and symptomatic treatment are usually involved to relieve this symptom, and in our study, it was found that patients who had taken TCM had a lower incidence of AEs. However, as for the serious AEs of high grades, we were not able to analyze them because of the lack of data on different degrees and types of side effects, for which we only analyzed the difference in the number of AEs.

We also perform network pharmacology to explore the specific effect of the TCM group on the GC treatment process. According to the results of the PPI and KEGG parts, the TCM group plus three core genes mentioned above and the relevant signaling pathway obtained by GO and KEGG enrichment analysis have more to do with the occurrence and development of malignant tumors. Current studies demonstrate that the level of NPM1 is associated with the promotion of endometrial cancer by circWHSC1 (32). In addition to being involved in inhibiting OC proliferation and apoptosis (33), HSP90AA1 has been shown to inhibit UCC cell invasion and the EMT phenotype (34). Furthermore, HSPA8 is considered to be another candidate biomarker for early diagnosis and treatment of EC in stage I (35), while the levels of plasma intima-media thickness plus serum high-density lipoprotein levels are considered to be a new marker of uterine fibroids (36). Frontier studies have been discussing the relationship between cardiovascular disease and cancer, and the results of our study could also provide new ideas for the development of this field.

NPM1 is a protein widely expressed in the nucleoli, which can travel between the nucleus and cytoplasm, participate in the assembly and transportation of ribosomal proteins, regulate centrosome replication, and regulate the expression of tumor suppressor ARF. Mutations in NPM1 push the protein to transfer from the nucleus to the cytoplasm, causing acute myeloid leukemia. However, Fan et al. (37) showed that in normal fallopian tubes, NPM1 protein was mainly distributed in the nucleus. The expression of NPM1 protein in HGSC was abnormal in the cytoplasm and significantly higher than that in normal oviduct tissue. Furthermore, APE1/Ref-1 interacts with NPM1 to control the DNA damage repair system (38). NPM1 also promotes UCC lymph node metastasis by participating in reprogramming fatty acid metabolism (39). Another study (40) demonstrated that inhibition of nuclear phosphine (NPM1/B23) restores the expression of estrogen receptor (ESR1/ERa) in endometrial cancer cells. This fully demonstrates the importance of this gene to GC.

Moreover, HSPA8 is a component of the HSP70 family expressing homologous proteins and is crucial to many cellular processes. In particular, its regulatory role in autophagy is decisive (41). Functionally, it binds to nascent peptides to promote correct folding. It also acts as an ATPase to break down cypermethrin-coated vesicles during the transport of membrane components through cells. According to a relevant study (42), HSPA8 can be used as a special diagnostic protein marker model for UCC. It was found that Hsp70 and Hsc70 are expressed in a unique and predominantly overlapping pattern in the cervix and endometrium as well (43). In both tissues, the highest levels of these two proteins were found in differentiated, nonproliferating epithelial cells. This means that the HSPA8 gene may also have a common expression in GC, but there is still a lack of relevant studies in this respect.

Furthermore, the HSP90AA1 gene is a core gene directly associated with GC. Functioning as a homologous dimer, the protein it encodes is an inducible molecular chaperone. The encoded proteins contribute to the correct folding of specific target proteins through the cochaperone-regulated ATPase activity. There is evidence (44) that HSP90A plays a key role in the crossroads between the Nanog-TCL1A axis and the multiple attack properties of immunoediting tumor cells. It stabilizes and enhances AKT activation through TCL1A, thereby contributing to the multi-invasive properties of NANOGhigh tumor cells. Importantly, HSP90 inhibition sensitized immune-refractory tumors to adoptive T-cell metastasis and PD-1 blockade and reactivated the immune cycle of tumor-responsive T cells. These results indicate that the HSP9OA-TCL1A-Akt pathway ignited by NANOG is a central molecular axis and potential target of immune-refractory tumors and has a high reference value for GC treatment.

The strengths of our study include our wide-ranging online searches of literature in both English and Chinese databases. We applied broad criteria for pooling studies. We conducted a meta-regression analysis to determine if specific preparation yielded differing effects over the broad group, and in several cases they did.

Some limitations may affect the drawn conclusion. First, there was a lack of large, multicenter, standardized RCTs, and the sample sizes of our included studies were mostly small or of moderate size. More RCTs with larger sample sizes are required to confirm these outcomes. Second, TCM’s individualized treatment and self-formulated prescription for inconsistent treatment with Chinese medicine may have led to heterogeneity in the meta-analysis, and none of them were able to provide definitive options for clinical treatment. Moreover, randomization methods, allocation concealment, and blinding are not clearly described in those included trials, which may result in inherent bias and exaggeration of the efficacy of the treatment group. In addition, our study induced a certain degree of heterogeneity due to the different classifications of tumors, pathologic stage of the tumor, the application of adjuvant therapy, and duration of treatment among included trials. Furthermore, almost all studies were conducted in China, which may lead to unavoidable regional bias. All these limitations may have resulted in insufficient evaluations of the outcome indicators. There are also some shortcomings in network pharmacology analysis. Due to the incomplete research on drug components and targets, the relevant data in the disease database are incomplete, leading to certain limitations and defects in the statistical results of network pharmacology in this paper. Also, the method of screening components of TCM by DL and OB values did not take the active ingredients into full consideration, which may affect the results. Furthermore, network pharmacology studies ignored the influence of component content of TCM on experimental results, and subsequent studies should focus on the relationship between content and curative effect. Moreover, network pharmacology is based on computer simulation analysis and molecular network data, whose statistical and predictive results need to be confirmed by specific experiments *in vivo* and *in vitro*. So we still need to take these compelling results with caution and this study should be carefully disseminated for clinical application. Our findings need to be further confirmed *via* more RCTs conducted in a rigorous manner.

All in all, we hope the findings of this analysis will provide a shred of helpful evidence for clinicians to formulate the best treatment strategy for patients with GC and also provide scientific clues for researchers in this field. Moreover, the methodological ideas of this paper are shown in **Supplementary Figure S13**.

## 5 Conclusion

In summary, our study indicates that TCM plus chemotherapy is highly effective and safe in the treatment of people with GC. TCM plus chemotherapy has the characteristics of multiple pathways, multiple components, and multiple targets in the treatment of GC and diseases. Our study will provide valuable evidence for further evaluation of TCM. Moreover, some limitations increased the risk of bias, which, to some extent, affects the credibility of the satisfactory result. Thus, to confirm these finding, more rigorous randomized controlled trials are needed.

## Data Availability Statement

The original contributions presented in the study are included in the article/**Supplementary Material**. Further inquiries can be directed to the corresponding author.

## Author Contributions

LZ, LS and JY contributed to the design and conception of the study. JY and NR carried out the collection and processing of data. LQ and GY finished the figure diagramming. ZZ and LY performed the data analysis and interpretation. NR and LY wrote the manuscript. LZ, LS and JY revised the manuscript, then LZ gave the final approval of manuscript.

## Funding

This study was financially subsidized by the Project funded by Plan Project of Zhejiang Province Medical and Health Science and Technology (LS, No. 2022RC215); China Postdoctoral Science Foundation (LS, No. 2021M702928); Young Elite Scientists Sponsorship Program by CAST (LS, No. 2021-QNRC2-B13); Research Project of Zhejiang Chinese Medical University (JY, No. 2021RCZXZK10); The Construction Fund of Medical Key Disciplines of Hangzhou (OO20200385); Research Project of Zhejiang Chinese Medical University (LZ, No.2022JKJNTZ40).

## Conflict of Interest

The authors declare that the research was conducted in the absence of any commercial or financial relationships that could be construed as a potential conflict of interest.

## Publisher’s Note

All claims expressed in this article are solely those of the authors and do not necessarily represent those of their affiliated organizations, or those of the publisher, the editors and the reviewers. Any product that may be evaluated in this article, or claim that may be made by its manufacturer, is not guaranteed or endorsed by the publisher.

## Abbreviations

TCM, traditional Chinese medicine; GC, gynecological cancer; CBM, Chinese biological medicine; CNKI, China National Knowledge Infrastructure databases; ORR, objective response rate; DCR, disease control rate; PD, progressive disease; QOL, quality of life; AEs, adverse events; RR, risk ratio; SMD, standardized mean difference; WMD, weighted mean difference; CIs, credible intervals; OC, ovarian cancer; PRISMA, Preferred Reporting Items for Systematic Reviews and Meta-Analysis; CR, complete response; PR, partial response; SD, stable disease; PD, progressive disease; VN, neoplasms; VC, vaginal cancer; UCC, uterine cervix cancer; EC, endometrial carcinoma; FTC, fallopian tube carcinoma; TCMSP, Traditional Chinese Medicine Systems Pharmacology Database and Analysis Platform; GO, gene ontology; KEGG, Kyoto Encyclopedia of Genes and Genomes; OB, oral bioavailability; DL, drug like, BC, betweenness centrality; BP, biological process; CC, cell composition; MF, molecular function; OS, overall survival; PFS, progression-free survival.
