# Supplementary material for: Exploring the Pharmacological Mechanism of the Effective Chinese Medicines Against Gynecological Cancer Based on Meta-Analysis Combined With Network Pharmacology Analysis
Source: Front Oncol. 2022 Jul 6;12:817772. doi: 10.3389/fonc.2022.817772 (PMC9298573; doi:10.3389/fonc.2022.817772)
Supplement: Supplementary file 1 [file DataSheet_1.docx]

***SUPPLEMENTARY MATERIAL***

1. **Supplementary Methods**

**Supplementary Method 1**

1. **Supplementary Figures**

**Supplementary Figure S1** – Sensitivity analysis of objective response rate in included RCTs

**Supplementary Figure S2** – Sensitivity analysis of disease control rate in included RCTs

**Supplementary Figure S3** – Sensitivity analysis of progressive disease in included RCTs **Supplementary Figure S4** – Sensitivity analysis of quality of life in included RCTs

**Supplementary Figure S5** – Sensitivity analysis of level of CD3+T in included RCTs

**Supplementary Figure S6** – Sensitivity analysis of level of CD4+T in included RCTs

**Supplementary Figure S7** – Sensitivity analysis of level of CD8+T in included RCTs

**Supplementary Figure S8** – Sensitivity analysis of level of CD4^+^/CD8^+^T in included RCTs

**Supplementary Figure S9** – Sensitivity analysis of level of incidence of adverse events in included RCTs

**Supplementary Figure S10** – GO enrichment analysis of biological process

**Supplementary Figure S11** – GO enrichment analysis of cell composition

**Supplementary Figure S12** – GO enrichment analysis of molecular function

**Supplementary Figure S13** – Technology roadmap for meta-analysis and network pharmacology analysis

**Supplementary Method 1**

The combined text and medical subject heading (MeSH) terms were cross-searched using MeSH and free word as follows: (Genital Neoplasms, Female [MeSH Terms] OR Gynecologic Neoplasm* [Title/Abstract] OR Female Genital Neoplasm [Title/Abstract] OR (neoplas* [Title/Abstract] OR tumor* [Title/Abstract] OR cancer* [Title/Abstract] OR malignan* [Title/Abstract] OR tumour* [Title/Abstract] OR carcinom* [Title/Abstract] OR adenocarcin* [Title/Abstract]) AND (ovar* [Title/Abstract] OR fallopian tube [Title/Abstract] OR vulva* [Title/Abstract] OR vagina* [Title/Abstract] OR cervi* [Title/Abstract] OR female genital [Title/Abstract] OR gynecologic [Title/Abstract]) AND (Medicine, Chinese Traditional [MeSH Terms] OR traditional medicine [Title/Abstract] OR traditional Chinese medicine [Title/Abstract] OR TCM [Title/Abstract] OR Chinese medicine [Title/Abstract] OR alternative medicine [Title/Abstract] OR complementary medicine [Title/Abstract] OR Drugs, Chinese Herbal [Title/Abstract] OR Chinese herbal medicine [Title/Abstract] OR herbal medicine [Title/Abstract] OR herbs [Title/Abstract] OR Chinese Herbal Drug* [Title/Abstract] OR Chinese Herbal Medicine* [Title/Abstract] OR Chinese Plant Extracts [Title/Abstract]) AND (Randomized Controlled Trial [Publication Type] OR Controlled Clinical Trial [Publication Type] OR Clinical Trials as Topic[Mesh:NoExp] OR randomized[Title/Abstract] OR placebo [Title/Abstract] OR randomly[Title/Abstract] OR trial[Title/Abstract]) NOT (Animals[Mesh] NOT Humans[Mesh])

**Supplementary Figure S1** – Sensitivity analysis of objective response rate in included RCTs.

**Supplementary Figure S2** – Sensitivity analysis of disease control rate in included RCTs.


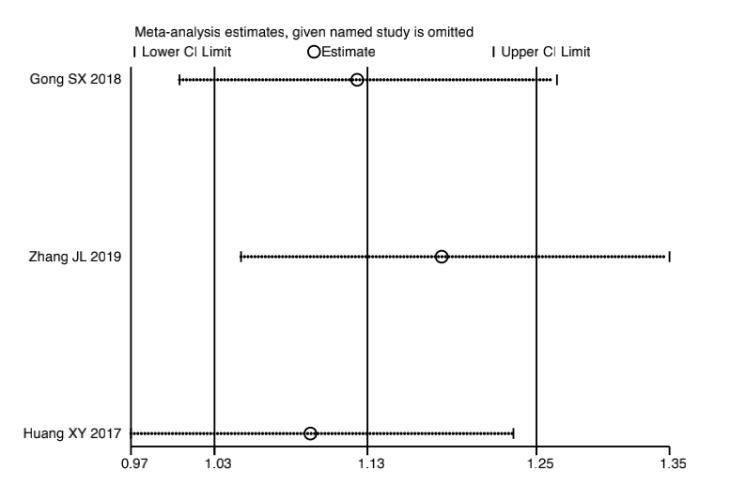


**Supplementary Figure S3** – Sensitivity analysis of progressive disease in included RCTs.

**Supplementary Figure S4** – Sensitivity analysis of quality of life in included RCTs.

**Supplementary Figure S5** – Sensitivity analysis of level of CD3+T in included RCTs.

**Supplementary Figure S6** – Sensitivity analysis of level of CD4+T in included RCTs.

**Supplementary Figure S7** – Sensitivity analysis of level of CD8+T in included RCTs.

**Supplementary Figure S8** – Sensitivity analysis of level of CD4^+^/CD8^+^T in included RCTs.

**Supplementary Figure S9** – Sensitivity analysis of level of incidence of adverse events in included RCTs.

**Supplementary Figure S10** – GO enrichment analysis of biological process.


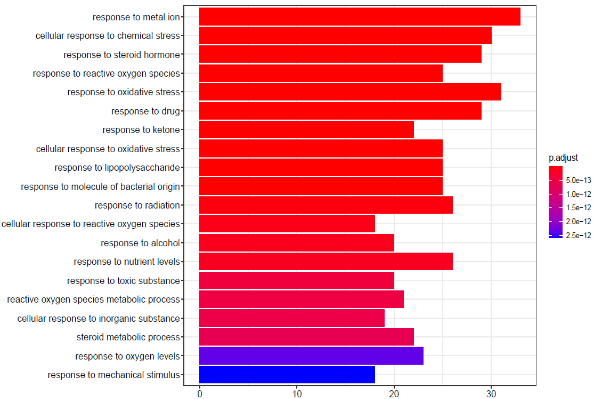


**Supplementary Figure S11** – GO enrichment analysis of cell composition.


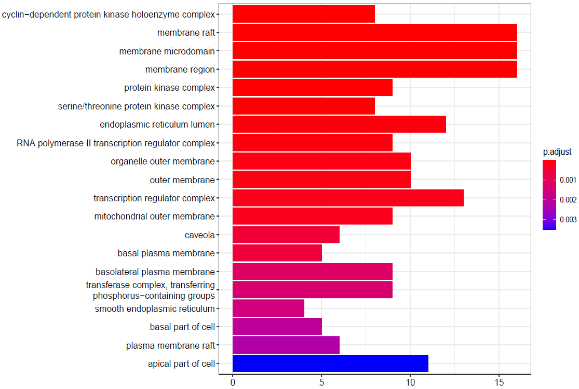


**Supplementary Figure S12** – GO enrichment analysis of molecular function.


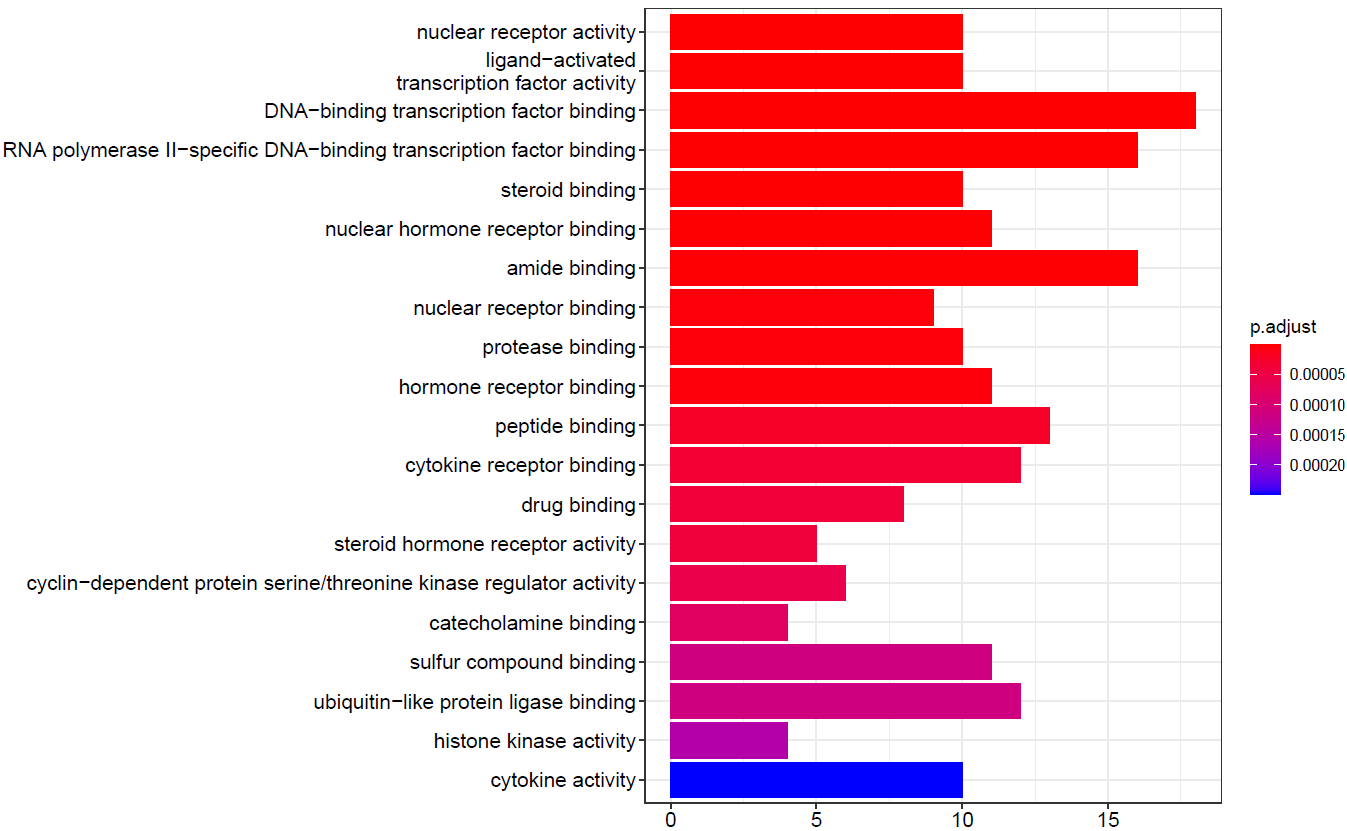


**Supplementary Figure S13** – Technology roadmap for meta-analysis and network pharmacology analysis.
